# Supplementary figures and images for: Hypomorphic mutation of the mouse Huntington’s disease gene orthologue
Source: PLoS Genet. 2019 Mar 21;15(3):e1007765. doi: 10.1371/journal.pgen.1007765 (PMC6445486; doi:10.1371/journal.pgen.1007765)

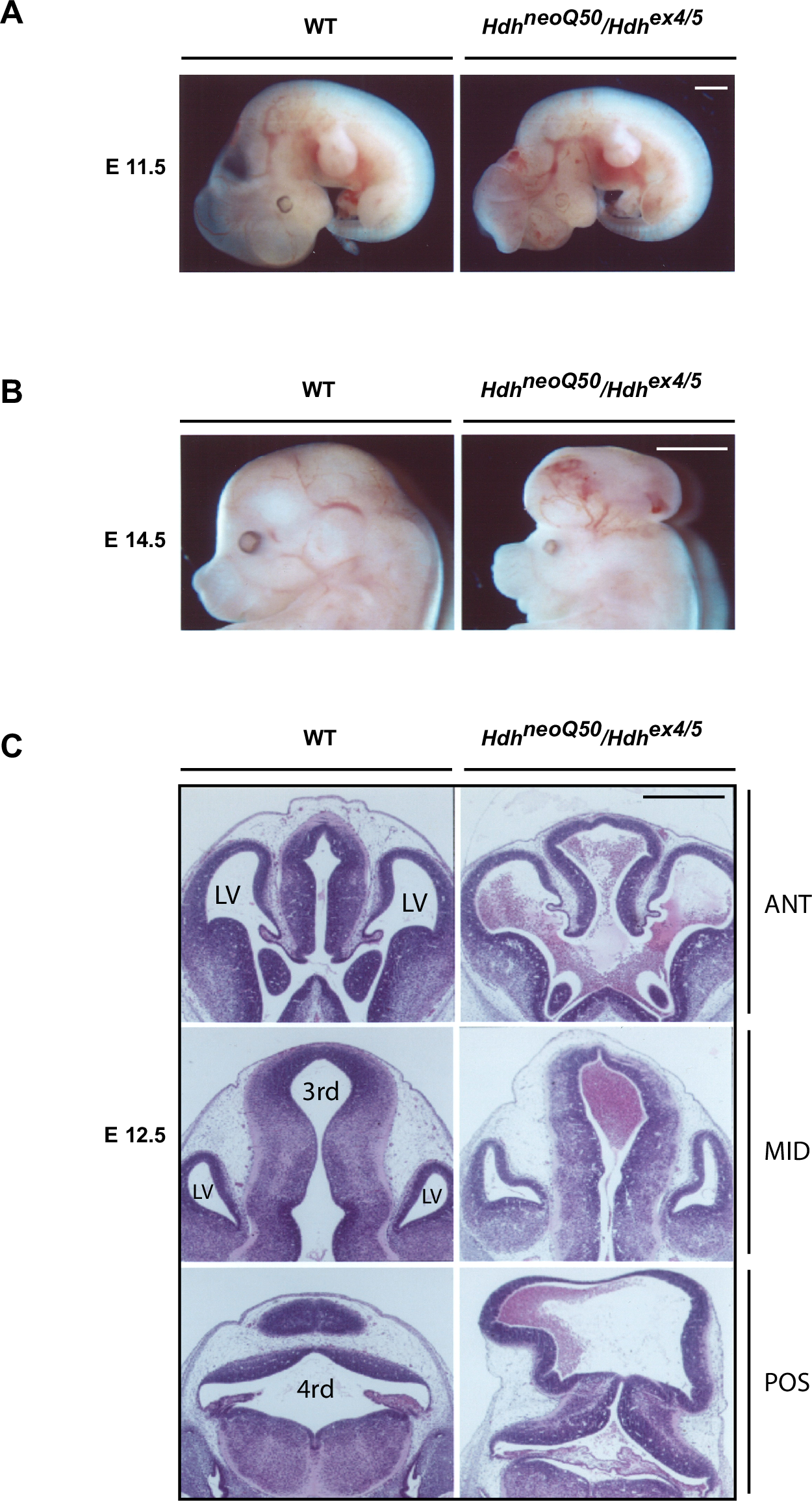

Supplement: S1 Fig — A-B) Representative pictures of WT and HdhneoQ50/Hdhex4/5 embryos at E11.5 and E14.5 developmental stages. Visible vasculature defects are present in animals, especially at the level of the brain. Scale bars = 1000μm. C) Representative pictures of coronal histological sections at the anterior (ANT), medial (MED) and posterior (POS) levels of HdhneoQ50/Hdhex4/5 embryos brains confirm enlarged ventricles with extensive blood accumulation. LV, lateral ventricles; 3rd, third ventricle, 4th fourth ventricle. Scale bar = 500μm. (TIF) [file pgen.1007765.s001.tif]

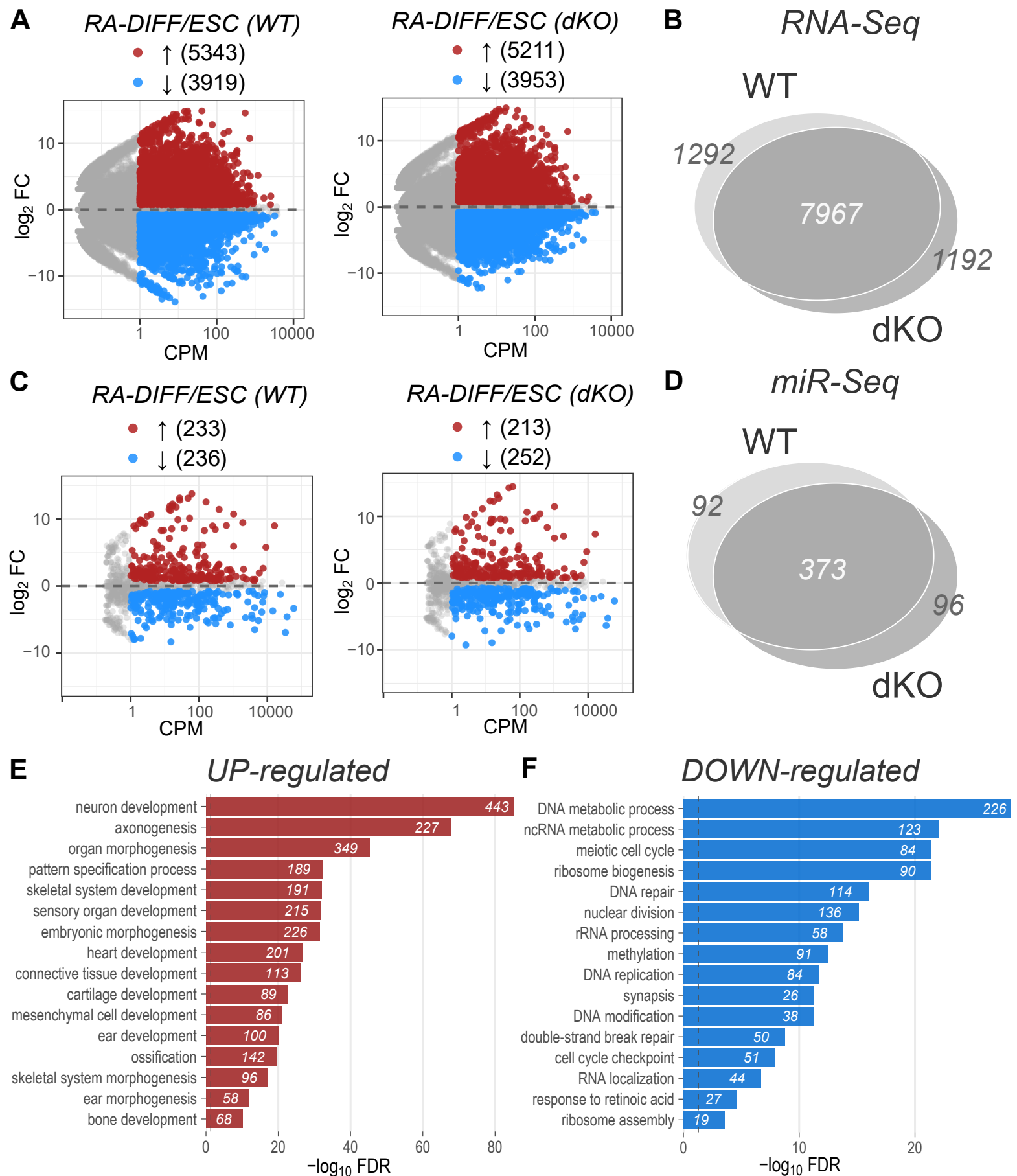

Supplement: S2 Fig — A) M (log ratio) and A (mean average) (MA) plot representations of mRNA-seq pairwise comparisons of RA-DIFF cells by wild-type (WT) or Hdhex4/5/ex4/5 Htt null (dKO) genotypes showing the average log10 signal (Counts Per Million—CPM) against the log2 Fold Change (FC) for each gene. Genes significantly up- regulated or down-regulated in the comparison are highlighted in red and blue, respectively. Numbers of differentially expressed genes are displayed (parenthesis). B) The Venn diagram reports the total number of genes that are commonly or specifically dysregulated during differentiation (transition from ESC to RA-DIFF), comparing cells with Htt wild-type (WT) or Htt-null (dKO) genotypes. C) MA plot representations of miRNA-seq pairwise comparisons. Legends, abbreviation and colors as in A). D) The Venn diagram reports the total number of miRNAs that are commonly or specifically changed during RA differentiation by wild-type (WT) or Hdhex4/5/ex4/5 Htt null (dKO) genotypes. E) Cluster 1 contains genes that are highly expressed in ESC and whose expression decreases during RA differentiation. Bar plots for this cluster report the most enriched GO-terms describing biological processes. The number of genes within each GO-term is also indicated (number within bars). F) Cluster 2 groups genes that are poorly expressed in ESC, but strongly upregulated during RA differentiation. Bar plots report the 5 most enriched GO terms associated with genes in Cluster 2. The number of genes within each GO-term is also indicated (number within bars). Genes belonging to Cluster 1 and 2 show similar behavior in cells with Htt wild-type (WT) or Htt-null (dKO) genotypes. (PDF) [file pgen.1007765.s002.pdf]

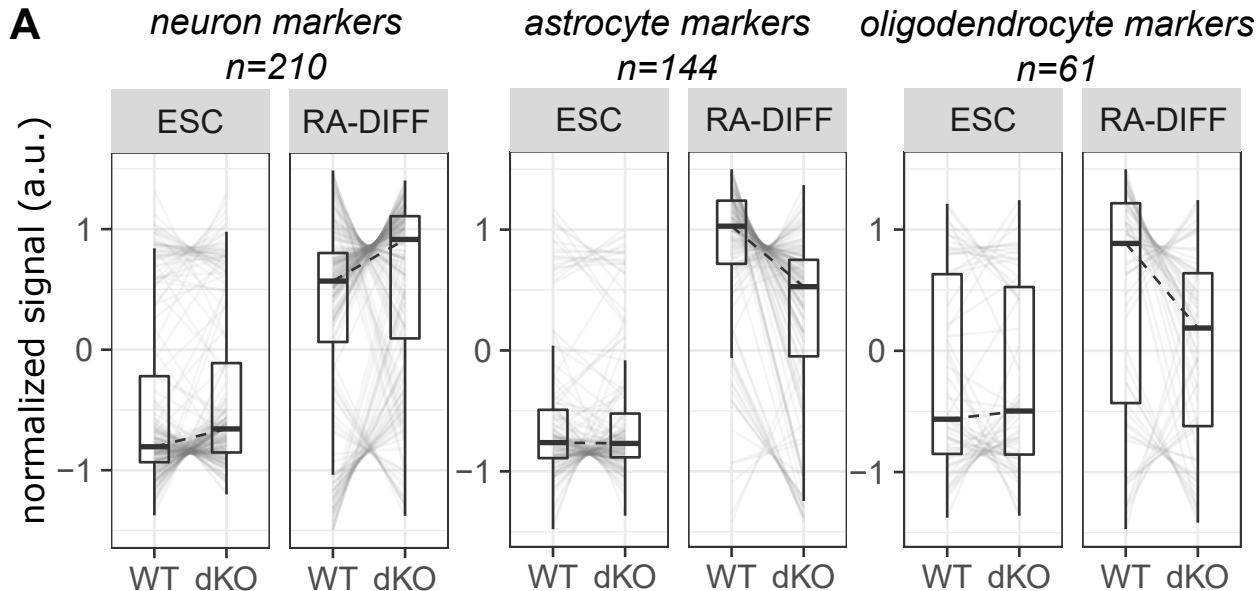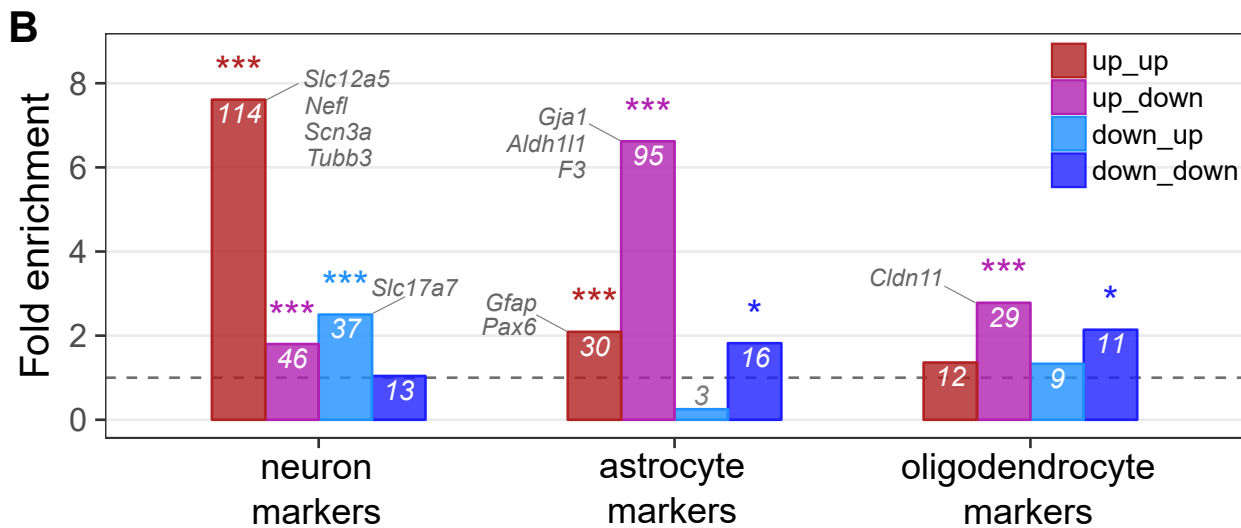

Supplement: S4 Fig — A) Expression trajectory plots describing variations in transcriptional levels of genes involved in neuron-glial specification during RA-DIFF: transcriptional changes for neuron markers, astrocyte markers, oligodendrocyte markers [24] between the two Htt genotypes (wild-type = WT and Htt-null = dKO) and two developmental stages (ESC and RA-DIFF) are shown. The number of genes defining each class of markers is indicated at the top of each plot. B) Enrichment bar plots depicting the enrichment values of neuron, astrocyte and oligodendrocyte markers among the 4 gene classes (up_up; up_down; down_down; down_up, as described in Fig 3A) of genes affected by RNA-differentiation and by Hdhex4/5/ex4/5 null mutation. The number of genes enriched in each of the 4 classes is indicated within each column. Statistical significance, measured by Fisher exact test is indicated by asterisks: (*) P<0.05, (***) P<0.01. (PDF) [file pgen.1007765.s004.pdf]
