# Supplementary material for: Hypomorphic mutation of the mouse Huntington’s disease gene orthologue
Source: PLoS Genet. 2019 Mar 21;15(3):e1007765. doi: 10.1371/journal.pgen.1007765 (PMC6445486; doi:10.1371/journal.pgen.1007765)
Supplement: S3 Fig — A) M (log ratio) and A (mean average) (MA) plot representations of mRNA-seq pairwise comparisons of Hdhex4/5/ex4/5 Htt null (dKO) versus wild-type (WT) genotypes in ESC and RA-diff cells showing the average log10 signal (Counts Per Million—CPM) against the log2 Fold Change (FC) for each gene. Genes significantly up- regulated or down-regulated in the comparison are highlighted in red and blue, respectively. Numbers of differentially expressed genes are displayed (parenthesis). B) The Venn diagram reports the total number of genes that are commonly or specifically dysregulated comparing cells with Htt wild-type (WT) or Htt-null (dKO) genotypes in ESC and RA-DIFF cells. C) MA plot representations of miRNA-seq pairwise comparisons. Legends, abbreviation and colors as in A). D) The Venn diagram reports the total number of miRNAs that are commonly or specifically dysregulated comparing cells with Htt wild-type (WT) or Htt-null (dKO) genotypes in ESC and RA-DIFF cells. E) Heatmap reports the top 25 most enriched Reactome and KEGG pathways associated with genes up and down-regulated in ESC and RA-DIFF cells in absence of huntingtin. The number of affected genes within each pathway is indicated (numbers in the cells). (PDF) [file pgen.1007765.s003.pdf]

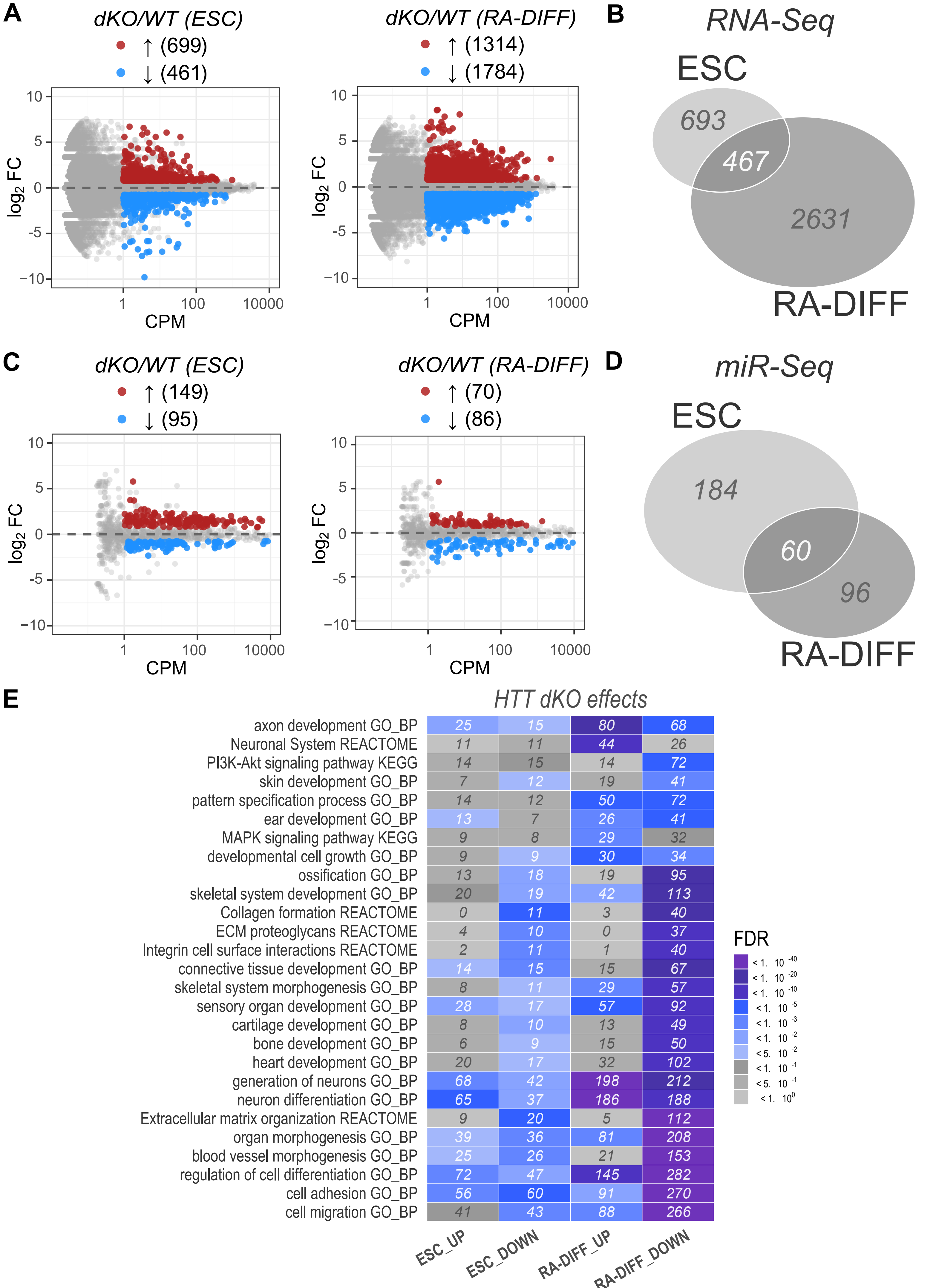
A
dKO/WT (ESC)
↑ (699)
↓ (461)
dKO/WT (RA-DIFF)
↑ (1314)
↓ (1784)
log2 FC
CPM
B
RNA-Seq
ESC
693
467
2631
RA-DIFF
C
dKO/WT (ESC)
↑ (149)
↓ (95)
dKO/WT (RA-DIFF)
↑ (70)
↓ (86)
log2 FC
CPM
D
miR-Seq
ESC
184
60
96
RA-DIFF
E
HTT dKO effects
axon development GO_BP 25 15 80 68
Neuronal System REACTOME 11 11 44 26
PI3K-Akt signaling pathway KEGG 14 15 14 72
skin development GO_BP 7 12 19 41
pattern specification process GO_BP 14 12 50 72
ear development GO_BP 13 7 26 41
MAPK signaling pathway KEGG 9 8 29 32
developmental cell growth GO_BP 9 9 30 34
ossification GO_BP 13 18 19 95
skeletal system development GO_BP 20 19 42 113
Collagen formation REACTOME 0 11 3 40
ECM proteoglycans REACTOME 4 10 0 37
Integrin cell surface interactions REACTOME 2 11 1 40
connective tissue development GO_BP 14 15 15 67
skeletal system morphogenesis GO_BP 8 11 29 57
sensory organ development GO_BP 28 17 57 92
cartilage development GO_BP 8 10 13 49
bone development GO_BP 6 9 15 50
heart development GO_BP 20 17 32 102
generation of neurons GO_BP 68 42 198 212
neuron differentiation GO_BP 65 37 186 188
Extracellular matrix organization REACTOME 9 20 5 112
organ morphogenesis GO_BP 39 36 81 208
blood vessel morphogenesis GO_BP 25 26 21 153
regulation of cell differentiation GO_BP 72 47 145 282
cell adhesion GO_BP 56 60 91 270
cell migration GO_BP 41 43 88 266
ESC_UP
ESC_DOWN
RA-DIFF_UP
RA-DIFF_DOWN
FDR
< 1. 10^-40
< 1. 10^-20
< 1. 10^-10
< 1. 10^-5
< 1. 10^-3
< 1. 10^-2
< 5. 10^-2
< 1. 10^-1
< 5. 10^-1
< 1. 10^0
