# Supplementary material for: Hypomorphic mutation of the mouse Huntington’s disease gene orthologue
Source: PLoS Genet. 2019 Mar 21;15(3):e1007765. doi: 10.1371/journal.pgen.1007765 (PMC6445486; doi:10.1371/journal.pgen.1007765)
Supplement: S1 Table — Table summarizes total number of raw and aligned reads for RNA-seq (A) and miRNA-seq (B) experiments. Data are related to Fig 6 and S2 and S3 Figs. (DOCX) [file pgen.1007765.s005.docx]

**Suppl. Table 1 related to Figure 6 and Suppl. Fig 2 and 3**

**Total number of aligned reads for RNA-seq (A) and miRNA-seq (B)**

A)


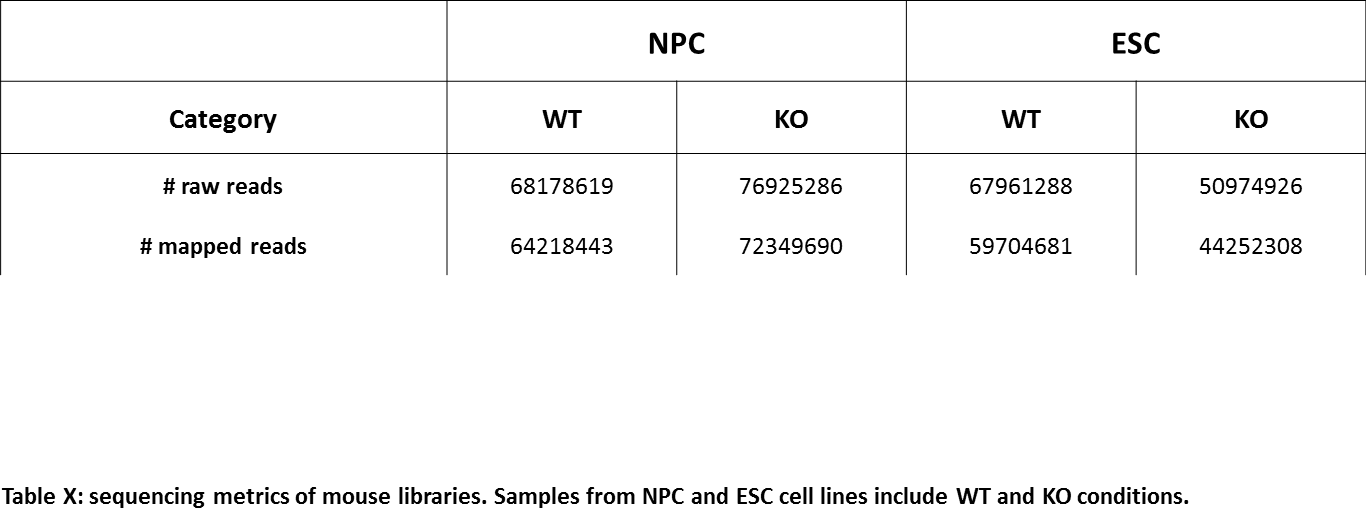


**RA-DIFF**

B)


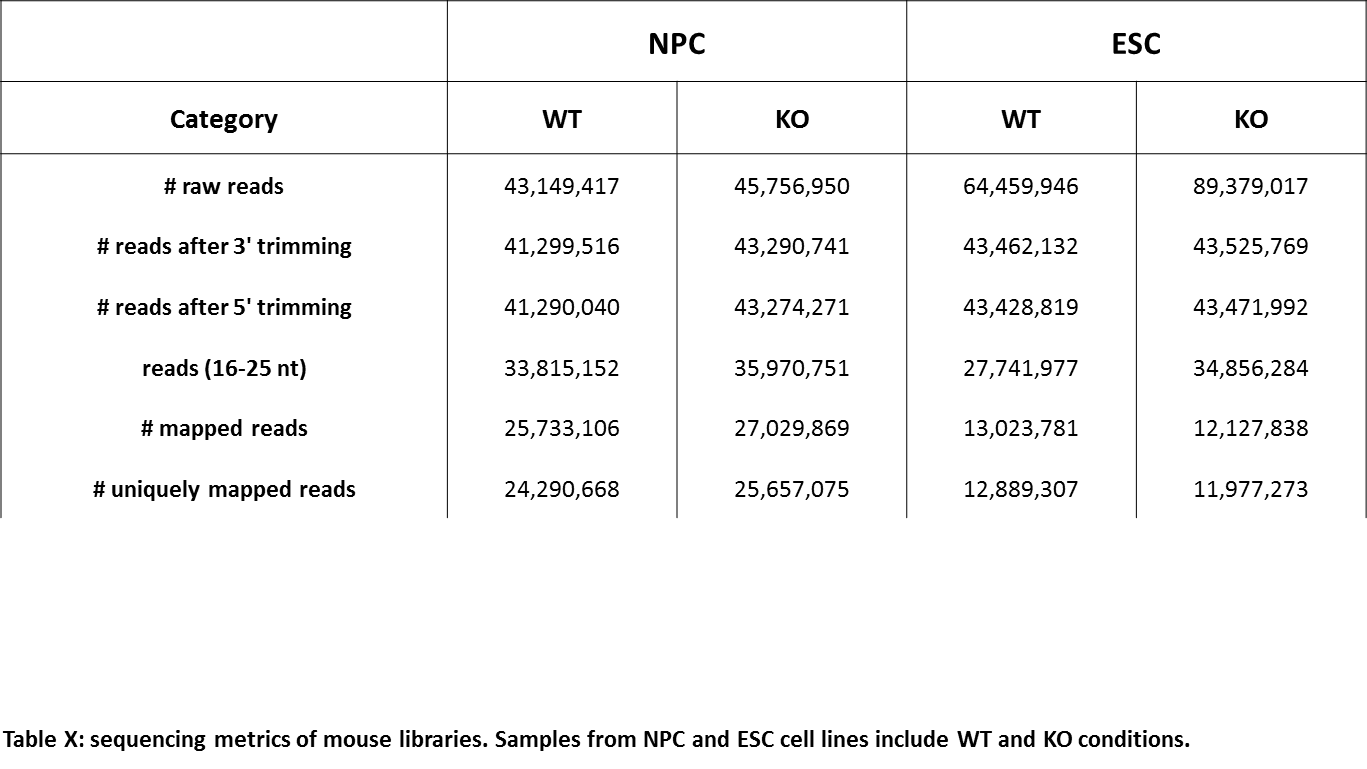


**RA-DIFF**
