# Supplementary material for: Hypomorphic mutation of the mouse Huntington’s disease gene orthologue
Source: PLoS Genet. 2019 Mar 21;15(3):e1007765. doi: 10.1371/journal.pgen.1007765 (PMC6445486; doi:10.1371/journal.pgen.1007765)
Supplement: S2 Table — Data are related to Fig 7 of the main text. (DOCX) [file pgen.1007765.s006.docx]

|  | Reference papers | | | Web-tools |
| --- | --- | --- | --- | --- |
| Body size | | 1. Kemper et al. Genome Biology 2012, 13:244 | <http://www.rgd.mcw.edu>  GO:0035264 Multicellular organism growth | |
| Skin | | 1. Jung H-J, et al. Molecular and Cellular Biology. 2014; 34(24):4534-4544 2. Richard G. 2004 American Journal of Medical Genetics Part C (Semin. Med. Genet.) 131C:32–44 | <http://www.rgd.mcw.edu>  MP:0003941 Abnormal skin development | |
| Skeleton | | 1. Lingjie Li et al. Cell Reports 2013; Volume 5, Issue 1, 3-12 2. Chen C et al. The Journal of Biological Databases and Curation. 2016; 2016:baw127 | <http://101.200.211.232/skeletongenetics/Analysis.php>  GO:0001501 Skeletal system development  <http://www.rgd.mcw.edu>  MP:0003036 Vertebral transformation | |
| Middle Ear | | 1. Mallo M. 1998.Int. J. Dev. Biol. 42, 11–22 2. Mallo M. 2001 Developmental Biology 231, 410–419 (2001) | <http://www.rgd.mcw.edu>  GO:0043583 Ear development, Middle ear morphogenesis | |
| Hematopoiesis | |  | <http://www.rgd.mcw.edu>  GO:0048534 Hematopoietic or lymphoid organ development | |

**Suppl. Table 2**

**Source description of the papers/webtools used to create the manually annotated genes’ lists for genes-associated-phenotypes**
